# The importance of transporters and cell polarization for the evaluation of human stem cell-derived hepatic cells

**DOI:** 10.1371/journal.pone.0227751

**Published:** 2020-01-23

**Authors:** György Török, Zsuzsa Erdei, Julianna Lilienberg, Ágota Apáti, László Homolya

**Affiliations:** Molecular Cell Biology Research Group, Institute of Enzymology, Research Centre for Natural Sciences, Hungarian Academy of Sciences Centre of Excellence, Budapest, Hungary; Universita degli Studi Di Cagliari, ITALY

## Abstract

One of the most promising applications of human pluripotent stem cells is their utilization for human-based pharmacological models. Despite the fact that membrane transporters expressed in the liver play pivotal role in various hepatic functions, thus far only little attention was devoted to the membrane transporter composition of the stem cell-derived liver models. In the present work, we have differentiated HUES9, a human embryonic stem cell line, toward the hepatic lineage, and monitored the expression levels of numerous differentiation marker and liver transporter genes with special focus on ABC transporters. In addition, the effect of bile acid treatment and polarizing culturing conditions on hepatic maturation has been assessed. We found that most transporter genes crucial for hepatic functions are markedly induced during hepatic differentiation; however, as regards the transporter composition the end-stage cells still exhibited dual, hepatocyte and cholangiocyte character. Although the bile acid treatment and sandwich culturing only slightly influenced the gene expressions, the stimulated cell polarization resulted in formation of bile canaliculi and proper localization of transporters. Our results point to the importance of membrane transporters in human stem cell-derived hepatic models and demonstrate the relevance of cell polarization in generation of applicable cellular models with correctly localized transporters. On the basis of our observations we suggest that conventional criteria for the evaluation of the quality of stem cell-derived hepatocyte-like cells ought to be augmented with additional elements, such as polarized and functional expression of hepatic transporters.

## Introduction

The liver is a complex organ with multiple physiological functions, composed of various cell types. Hepatocytes constitute the majority (78%) of the liver volume; however, other cell types, such as biliary epithelial cells (cholangiocytes), endothelial cells, and Kupffer cells, the liver-resident macrophages are also important components of the liver [1]. Hepatocytes are responsible for most of the liver functions, such as drug detoxification, cholesterol metabolism, bile production, albumin secretion, glycogen storage, etc. The tissue architecture of the liver is complex and crucial for hepatic functions. Hepatocytes are in contact with the blood through their basolateral membrane, allowing either the cellular uptake of various molecules, such as lipids, proteins, and xenobiotics, or the secretion of different products, such as albumin and clotting factors, into the blood stream. Apical surfaces of adjacent hepatocytes form a tube-like structure, the bile canaliculus, which is sealed with tight junctions, ensuring complete separation of the serosal and canalicular spaces. Hepatocytes secrete bile constituents into the canaliculi, as well as excrete endo- and xenobiotics into the bile.

Membrane transporters play pivotal role in this polarized structure, and their proper localization is critical for normal hepatic functions. In fact, transporters control the vectorial movement of different substances into, from, and across the hepatocytes. The bile secretion is exclusively driven by ABC transporters residing in apical membrane. ABCB11 or bile salt export pump (BSEP) is responsible for the transport of bile acids to the canalicular lumen [2,3], whereas ABCB4 (MDR3) flips phosphatidylcholine (PC) to the outer leaflet of the membrane, resulting PC efflux into the bile [4]. The ABCG5/G8 heterodimer is proposed to be responsible for the canalicular transport of cholesterol [5,6].

Transport processes are also critical for detoxification. Basolateral uptake transporters control the delivery of various molecules into the hepatocytes, where these compounds are metabolized and secreted into the bile. The Na^+^-taurocholate cotransporting polypeptide (NTCP) transports mainly bile acids from the blood to the hepatocytes [7,8], whereas the organic anion-transporting polypeptides (OATPs), such as OATP1B1 and OATP1B3, mediate uptake of large, non-polar drugs and hormones [9–12]. This step is also termed Phase 0 of drug elimination. The unwanted compounds are then oxidized and conjugated (Phases 1 and 2). Efflux transporters, residing in the canalicular membrane, such as ABCB1 (MDR1), ABCC2 (MRP2), and ABCG2, are responsible for biliary excretion of endo- and xenobiotics (Phase 3) [13–15]. A dissimilar set of ABCC proteins including ABCC1 (MRP1), ABCC3-6 (MRP3-6) transporters are located in the basolateral membrane; and many of them are thought to serve as overflow systems for the elimination of toxic compounds, when the canalicular secretions is not sufficient [16–20].

Proper cellular model systems, which can reliably reproduce these hepatic functions, are required for both basic research and pharmacological screenings. Currently available models for prediction of drug metabolism and transport include cell lines of human hepatocellular carcinoma origin, such as Huh-7, HepG2, and HepaRG, as well as animal-based models, such as primary hepatocytes isolated from of mice or rats [21–24]. The model cells of cancerous origin can only partially polarize, and their applicability is restricted, since they may not reproduce the hepatic functions, especially those that require polarized architecture. Utilization of primary cells from rodents is also limited, since they are not suitable for large-scale screening, and the interspecies differences raise numerous ambiguities. Lately, extensive research on human stem cells has opened new perspectives and provided encouraging results on generation of various human cell types, including hepatocytes. Hepatocyte-like cells (HLCs) differentiated from human pluripotent stem cells may thus represent an alternative for drug toxicity screening models [25–27]. Despite their pivotal role in hepatic functions, such as detoxification and bile secretion, thus far only very modest attention was paid to membrane transporters in stem cell-derived pharmacological models of liver cells.

In a previous study, we differentiated human embryonic cell lines, HUES1 and HUES9 toward the hepatic lineage, and characterized their progeny cells in terms of tight junction components [28]. In the present work, we provide a comprehensive analysis of hepatic differentiation of HUES9 cells, where the progress of differentiation was monitored by functional assays, as well as by a complex expression profiling with a special emphasis on the transporter composition of the differentiated cells. Our study also encompasses examination of the effect of different culturing conditions, such as conditioning with bile acids and sandwich culturing, on the hepatic differentiation of human embryonic stem cells.

## Materials and methods

### Cell culturing

HepG2 (HB-8065) cells obtained from ATCC were maintained at 37°C and in 5% CO_2_ atmosphere in DMEM/F-12 1:1 (Gibco) medium supplemented with 10% FBS, 200 mM L-glutamine (Gibco), and 1% penicillin/streptomycin (Gibco). The cells were passaged at 90% confluency using trypsin (Gibco). Terminally differentiated HepaRG cells were provided by Biopredic International for this study and maintained according to the manufacturer’s instructions. The HUES9 human embryonic stem cell line, having a normal karyotype, was a gift from Dr. Douglas Melton (Harvard University), and maintained in accordance with the recommended culturing protocol [29].

### Hepatic differentiation of HUES9

The differentiation protocol of stem cells toward the hepatic lineage was published in a previous paper [30]. For sandwich culturing, the differentiating HUES9 cells were overlaid with rat tail Collagen-I (0.325 mg/mL in D-MEM, Sigma), or growth factor-reduced Matrigel (0.25 mg/mL or 0.05 mg/mL in DMEM/F12 1:1 media, Corning) on day 13. For overlaying, the media were removed from the cells and replaced with the collagen or Matrigel mixture for 30 or 45 minutes, respectively. Following this, the culturing media were re-added, and the samples were handled in the same manner as the uncover cultures.

To study the effect of a bile acid on hepatic differentiation, in the indicated cases, the cells were subjected to 20 μM tauroursodeoxycholic acid (TUDCA obtained from Sigma) for the final 48 hours of differentiation (days 20–22).

### Functional assays of differentiated cells

Supernatants were collected form the cells at the end of each differentiation stage, 24 hours after medium change. The albumin level of samples was measured by human albumin ELISA kit (Assaypro), while the urea content was assessed by DIUR-500 kit (QuantiChrom) in accordance with manufacturers’ instructions. In parallel, the total protein content of each culture collected in Laemmli sample buffer was determined by Lowry method [31]. The secreted albumin and urea were normalized to the total protein content of each sample.

### Cell proliferation assay by EdU incorporation

The proliferating cells in the HLC cultures were determined using Click-iT Plus EdU imaging kit (Thermo Fisher Scientific). Pluripotent HUES9 cells and their differentiated progenies on day 21 were incubated with EdU for 24 hours, then fixed, permeabilized, and stained in accordance with the manufacturer’s instructions. The images of the stained samples were acquired and analyzed by ImageXpress Micro (Molecular Devices) high content analysis device.

### Immunocytochemistry

For immunofluorescence staining, HUES9 cells were seeded onto Imaging dishes (Zellkontakt) previously coated with Matrigel (50 μg/mL), and differentiated as described above. The cells on day 2, 12, 17, 22 were fixed and permeabilized with 4% paraformaldehyde in PBS for 15 min, then with ice cold methanol for an additional 15 min. Following a 1 hour blocking procedure with Dulbecco’s modified PBS containing 2 mg/mL BSA, 1% fish gelatin, 0.1% Triton X-100, and 5% goat serum (pH 7.2), the cells were subjected to primary antibodies against HNF4α (1:100, Abcam), alpha-fetoprotein (AFP, 1:200, Abcam), albumin (1:500, Abcam), KRT18 (1:100, Abcam), KRT19 (1:250, Abcam), ABCG2 (1:250, BD Biosciences), NTCP (1:100, Boster), or MRP2 (1:20, Abcam) for 2 hours. AlexaFluor-488-conjugated anti-mouse and AlexaFluor-594-conjugated anti-rabbit IgG secondary antibodies were used (1:250, Thermo Fisher Scientific). The cell nuclei were stained with 1 μM DAPI in DPBS for 10 min. The blue, green, and red fluorescence images of stained samples were acquired by an Olympus FV500-IX confocal laser scanning microscope using a PLAPO 60× (1.4) oil immersion objective (Olympus) at 405, 488 and 543 nm excitations, respectively.

HepG2 and terminally differentiated HepaRG cells were seeded onto Matrigel-coated (50 μg/mL) Imaging dishes (Zellkontakt), cultured for 24h, then overlaid with Matrigel (0.25 mg/mL in DMEM/F12 1:1 media, Corning), and maintained in accordance with the manufacturers’ protocol for an additional week. The cells were fixed and blocked, then stained with anti-occludin primary antibody (1:200, Abcam), AlexaFluor-594-conjugated anti-rabbit IgG secondary antibody (1:250, Thermo Fisher Scientific), and DAPI as described above. Differentiated HLCs grown in monolayer or sandwich culturing configuration were fixed, blocked, and then stained with anti-claudin-5 primary antibody (1:120, Invitrogen), AlexaFluor-594-conjugated anti-mouse IgG secondary antibody (1:250, Thermo Fisher Scientific), and DAPI as described above. The images were acquired by a Zeiss LSM710 confocal laser scanning microscope using a PlanApochromat 40× (1.4) oil immersion objective (Zeiss) at 405, and 546 nm excitations.

### CDCFDA extrusion assay

The 5-(and-6)-Carboxy-2',7'-Dichlorofluorescein Diacetate (CDCFDA, Thermo Fisher Scientific, C369) staining was carried out on differentiated (d22) HLCs, HepG2, and HepaRG cells grown on imaging chambers (Zellkontakt). The cultures were incubated with serum free media containing 2μM CDCFDA for 30 minutes at 37°C. Subsequently the samples were washed twice with pre-warmed PBS; the green fluorescence was visualized by Leica DM IL LED wide-field inverted fluorescence microscope using a HCX PL Fluotar 40× (0.75) dry objective (Leica).

### Gene expression analysis

Total RNA was extracted from HUES9 cells and their differentiated derivatives, as well as from control samples, such as HepG2 with passage number <5 (HepG2-LP) or >25 (HepG2-HP), and HepaRG cells using TRIzol reagent (Invitrogen), and then purified in accordance with the manufacturer’s instructions. Fetal and adult liver total RNA was purchased from Agilent technologies. cDNA synthesis was performed from 1μg total RNA using SuperScript VILO cDNS synthesis kit (Invitrogen). After cDNA synthesis, the samples were run on an own designed 384 well TaqMan Low-Density Array (TLDA) card (Life Technologies), using ABI Prism 7900HT machine. The TLDA card contained 8 reference genes, 36 lineage markers, and 47 membrane transporters, including 40 ABC protein genes (S1 Table). The expression of each gene of interest was normalized to the average expression of the 5 most stable reference genes [32] (dCt method). The relative expression values were calculated by ddCt method, and then fold changes were calculated (S1 Table). Principal Component Analysis (PCA) and cluster analysis (UPGMA algorithm) [33] were performed based on the normalized gene expressions (dCt values) of each sample, using PAST software [34]. The results represent of average values of 2 array runs each containing pooled cDNA of two biological replicates. The expression levels of CYP3A4 (assay ID: Hs00604506_m1) were determined in a separate set of experiments, and analyzed as described above. These results represent a single array run of pooled cDNA samples of two biological replicates.

Individual qPCRs were carried out using TaqMan assays (Life Technologies) for detecting total mRNA of HNF4, AFP, ALB, ABCG2, ABCB11 (assay IDs are shown in S1 Table), as well as the ribosomal protein, RPLP0 (assay ID: Hs99999902_m1). The latter was used as internal control for quantification. Relative mRNA levels were calculated by using the 2^-dCt^ method. The assays were run and analyzed by using the StepOnePlus real-time PCR system (Life Technologies), according to the manufacturer’s instructions.

## Results

### Functional characterization of human embryonic stem cell-derived hepatic cells

**Fig 1:**
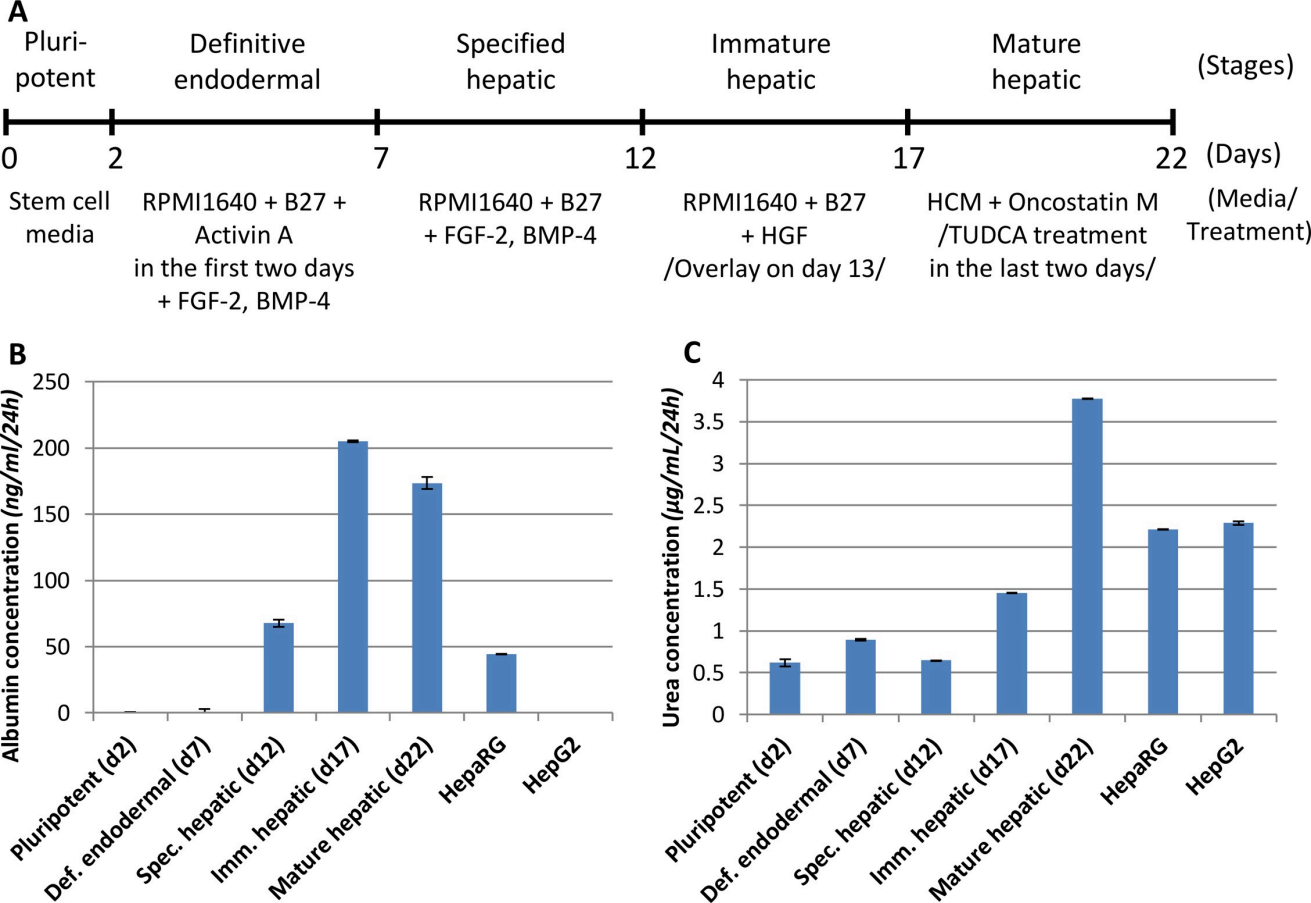
Hepatic differentiation of HUES9 stem cells. A: Schematic representation of the differentiation process indicating the various stages, the duration of each phase, the culturing media, and the different treatments applied. B, C: Albumin and urea secretion from the cultures during hepatic differentiation. The supernatants were collected 24 hours after medium change; the concentrations were measured and normalized to the total protein of each sample. As controls HepaRG and HepG2 cells were used. The error bars represent S.E.M. (n = 4).

The main goal of our study was to characterize of hepatic differentiation of human embryonic stem cells in terms of transporter expression and function. Since HUES9 stem cell line exhibited the best hepatic differentiation potential [28], we used this cell line in the present study. The cells were differentiated toward the hepatic lineage using a highly efficient, 22-day multi-step differentiation protocol consisting of 5 stages, i.e., pluripotent, definitive endodermal, specified hepatic, immature hepatic, and mature hepatic phases (Fig 1A). The progress of differentiation was monitored at the end of each stage.

A widely-accepted hallmark of *in vitro* hepatic differentiation of pluripotent cells is appearance of typical liver secretions in the medium. Therefore, we monitored the concentration of the secreted albumin and urea in the supernatants during hepatic differentiation. HepaRG and HepG2 cell lines were used as references. It should be noted that these cancerous cell lines are not ideal representation of hepatocytes, but they are widely used as hepatic model cells. During differentiation albumin was first detected at the endodermal stage and its level was gradually increased at subsequent stages (Fig 1B). The highest albumin concentrations were observed at the immature and mature hepatic stages, which levels greatly exceeded that was produced by HepaRG and HepG2, the latter of which hardly produced any albumin. It is noteworthy that HepG2 cells, as many cell lines of cancerous origin, are genetically instable, and their albumin secretion was greatly influenced by the passage number as demonstrated in S1 Fig. Urea was present in the supernatants from the beginning of differentiation, and its concentration elevated only in the last 10 days (Fig 1C). Urea levels in the medium of both control cells were relatively high, but stem cell-derived mature hepatic cells surpassed them. Urea secretion of HepG2 cells did not vary substantially with the passage number (S1 Fig).

**Fig 2:**
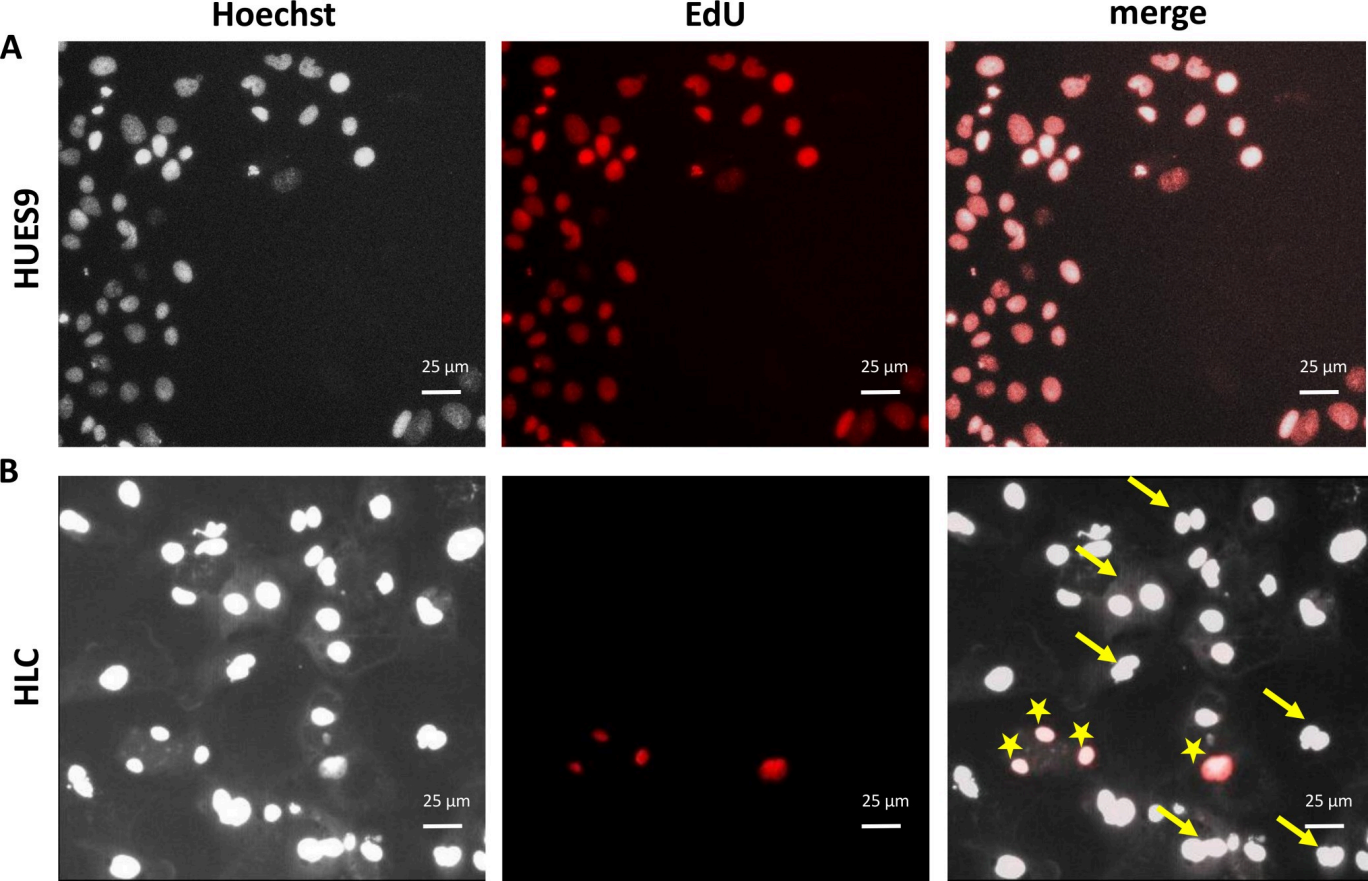
Assessment of cell proliferation in HUES9 and differentiated HLC cultures. A: Parallel Hoechst (left) and EdU (middle) staining of cell nuclei demonstrates that every cell in the HUES9 culture is proliferating (100% overlap). B: In the mature HLC culture, however, only a few cells undergo division. Hoechst staining also revealed numerous binucleated cells indicated by yellow arrows in the composite view (right panel), whereas EdU-positive cell are marked with asterisks.

Low proliferation rate and the presence of binucleated cells in the cultures are also implications of differentiated HLCs. To determine the proliferation rate at the final stage of differentiation, an Edu-based cell proliferation assay was performed. In the last 24 hours, only 7.15 ± 4.5% of the cells divided, whereas the figure in the pluripotent stage was 100% (Fig 2). At the final stage, numerous binucleated cells were observed, as indicated by yellow arrows in Fig 2B (right panel).

### Immunostaining of differentiated cells

**Fig 3:**
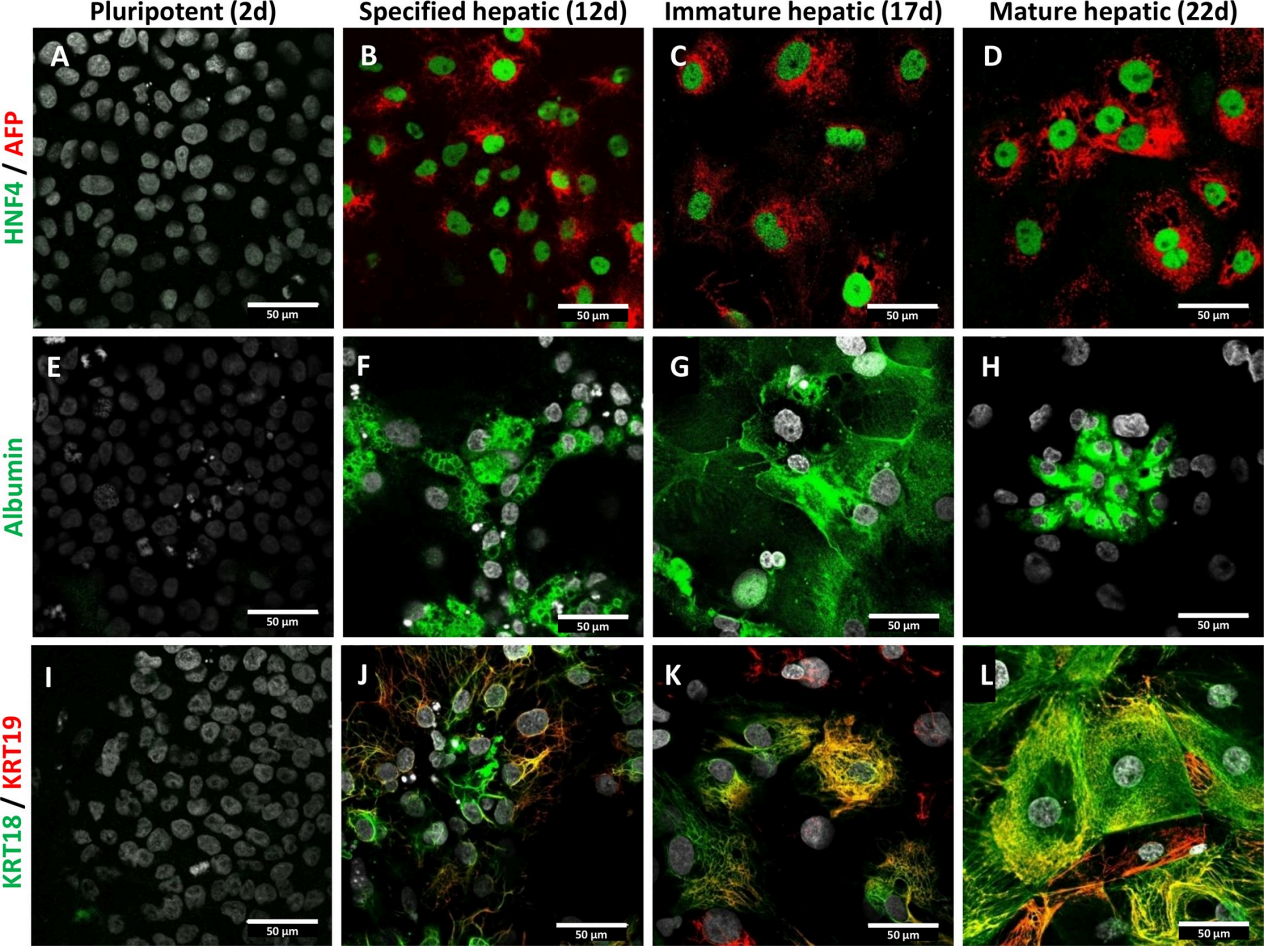
Expression of liver-specific markers during hepatic differentiation of HUES9 cells. A-D: Representative images demonstrate the expression pattern of HNF4α (green) and AFP (red) at different stages of differentiation as indicated at the top. Expression of albumin expression (green) is shown in Panels E-H, whereas expression of KRT18 (green) and KRT19 (red) are indicated in Panels I-L. All employed antibodies were specific to human antigens. The cell nuclei stained with DAPI are shown in grey. Scale bars represent 50 μm.

For further characterization, immunofluorescence staining of typical markers of differentiating liver cells was performed. The HNF4α expression is critical for hepatocyte development; therefore, its presence in differentiating cells is a key marker for hepatic differentiation. At the pluripotent stage, HNF4α was not detected; however, at later stages most of the cells expressed this transcription factor (Fig 3A–3D). Similar expression pattern was observed for AFP, which represents a fetal hepatocyte marker (Fig 3A–3D), indicating that the cells are committed to hepatic differentiation. Albumin was expressed from relatively early point (specified hepatic stage) throughout the differentiation (Fig 3E–3H). Interestingly, albumin was concentrated in secretory vesicles at the end of differentiation, which is in concert with the albumin secretion measurements (Fig 1B). Two keratins, the hepatocyte marker KRT18, as well as the cholangiocyte and hepatoblast marker KRT19 were also assessed in the differentiating cells (Fig 3I–3L). Most prominent expression of these proteins was observed at the final, mature hepatic stage. In the differentiated cultures, numerous cells were stained for either KRT18 or KRT19, a significant portion of the cells showed, however, double positivity.

### Gene expression analysis of hepatic differentiation

To monitor the changes in the gene expression patterns during hepatic differentiation, we designed a special, custom-made TaqMan array. This array contained probes not only for the usual lineage marker genes, such as AFP, albumin, HNF4α, keratins 18 and 19, etc., but also for genes encoding for metabolizing enzymes and transporter proteins, including ABC transporters. To diminish culture to culture variances and to observe general tendencies, mRNA profiling was performed with pooled samples obtained from two independent differentiations.

Since one of the goals of our study was to compare the human stem cell-derived hepatocyte-like cells with the commonly used human hepatic model cells, in addition stem cell-derived samples, four reference mRNA samples, including fetal and adult liver, as well as HepG2 and HepaRG cells were investigated. HepaRG cells have recently become prominent in pharmacological studies; however, the HepG2 cell line was formerly the most widely used model in liver-related investigations. Because of their genetic instability, cell lines with cancerous origin tend to undergo alteration with longer-term culturing. Therefore, we included samples of HepG2 cells with low (<5) and high (>25) passage number (denoted as HepG2-LP and HepG2-HP, respectively).

**Fig 4:**
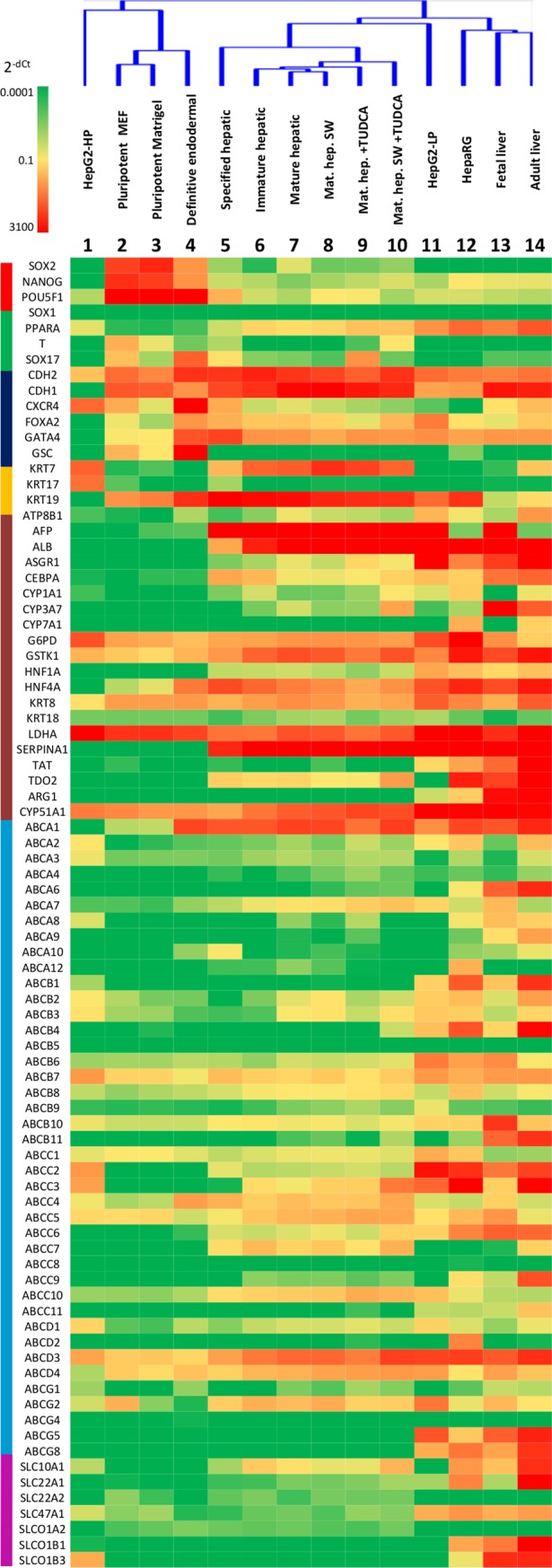
mRNA expression profiling and cluster analysis of hepatic cells differentiated from HUES9 cells, comparison with various liver cell models. A heat map was generated on the basis of mRNA expressions determined by a custom-designed TLDA card, where the expression levels relative to 5 stable reference genes are color-coded as indicated on the top left scale bar. Colors at the left indicate the various groups of genes as follows: red–pluripotency, green–lineage, navy–early hepatic, yellow–cholangiocyte, brown–hepatocyte markers; blue–ABC transporters, magenta–uptake transporters. Detailed description of the studied genes and the fold changes in mRNA expressions can be found in S1 Table. The dendrogram at the top demonstrate the hierarchical cluster analysis. The sample are as follows: 1 –HepG2 cells with high passage number, 2 –HUES9 cells grown on feeder cells, 3 –HUES9 cells grown on Matrigel, 4–7 –HUES9-derived cells progressively differentiated toward the hepatic lineage, 8 –stem cell-derived mature hepatic cells grown under sandwich culturing conditions, 9 –stem cell-derived mature hepatic cells treated with TUDCA, 10 –sandwich-cultured mature hepatic cells treated with TUDCA, 11 –HepG2 cell with high passage number, 12 –HepaRG cells, 13 and 14 –RNA samples from fetal and adult liver, respectively.

To evaluate the alterations in mRNA expressions during hepatic differentiation, a heat map was generated on the basis of the dCt values (Fig 4). An alternative evaluation approach was, when the fold changes in the expression levels relative to the mature hepatic stage (or relative to the adult liver sample) were calculated (S1 Table). The mRNA level of pluripotency genes, such as SOX2, NANOG, and POU5F1, were rapidly declined during the differentiation as documented in Columns 2–7 of Fig 4. For comparison, the levels of these genes were low in the reference cells (Columns 1, and 11–14). The expression of hepatic differentiation markers, including AFP, ALB, ASGR1, HNF1A, and AAT, were markedly induced indicating hepatic commitment of the differentiated cells. As expected, endoderm and early hepatic markers, such as SOX17, CXCR4, GATA4, and GSC, exhibited a transient elevation in their expression levels. Interestingly, the mRNA levels of some biliary markers (KRT7, KRT19) were also elevated; however, the KRT17 level was hardly detectable throughout the differentiation process.

Two of the studied monooxygenases, CYP1A1 and CYP3A7 were induced in the later phase of differentiation starting from specific and immature hepatic stage, respectively, while CYP7A1 was not detected in either the stem cell-derived HLCs or HepG2 cells, observed only in the HepaRG and the adult liver samples. The expression of CYP3A4 was also studied in a separate set of experiments. This enzyme only appeared in the final, mature hepatic phase (S2 Fig), which is in accordance with the fact that CYP3A4 is an adult form of hepatic CYP enzymes. As compared to the level found in the mature HLCs, the expression of CYP3A4 was similar in HepG2-LP cells, about 4-fold and 300-fold in HepaRG cells and the adult liver sample, respectively. This CYP enzyme was not detected in HepG2-HP cells and the fetal liver sample.

Out of the studied uptake transporters, SLC10A1 (NTCP) and SLC22A1 (OCT1) were markedly induced in the stem-cell derived hepatic cells. Interestingly, SLC47A1 (MATE1), and SLCO1A2 (OATP1A2) were expressed in the human pluripotent stem cells, and their level remained basically unchanged during differentiation. Similarly, SLC22A2 (OCT2) was also present in the HUES9 cells; its expression level transiently declined, and later regained in the course of hepatic differentiation. However, the expression of two important uptake transporters in the liver, SLCO1B1 (OATP1B1) and SLCO1B3 (OATP1B3) remained undetectable throughout the differentiation.

To explore culture to culture variations, few selected genes in HLCs derived from three independent differentiations were also assessed by qPCR. For comparison, HUES9, HepG2-LP and adult liver mRNA samples were used. Expression levels of albumin, AFP, and HNF4 were alike in HLCs of different differentiations (S3 Fig), which observation is in good agreement with our previous results obtained with HUES9-derived HLCs [28]. However, ABCB11 and ABCG2 were not induced in one out of three cultures, although their mRNA levels were similar, when expressed. This observation implies that assessment of the usual hepatic markers alone is not sufficient to evaluate the quality of the differentiated cells.

### The effect of sandwich culturing and tauroursodeoxycholic acid

Previous studies demonstrated that 3D and so-called sandwich culturing conditions facilitate polarization of hepatocytes and have a positive effect on the hepatic differentiation [35–39]. It has also been reported that bile acids can elevate the expression of liver specific transporters, and enhance their membrane localization [40–42]. The cultures differentiated from HUES9 cells toward the hepatic lineage were overlaid with Matrigel on day 13, and compared with the cultures, which were not overlaid (monolayers). To investigate the effect of bile acids, both monolayers and sandwich cultured cells were subjected to TUDCA on day 20. As gene expression analysis demonstrated, Matrigel sandwich culture condition had only a minor effect on the mRNA expressions. Only keratin 7 and SLC22A2 (OCT2) expressions were elevated about 2-fold in response to Matrigel overlay (S1 Table). Neither TUDCA treatment had a major effect on the expression profile of the stem cell-derived hepatic cells. Only the endodermal marker SOX17 showed elevated expression in the presence of TUDCA. However, the combination of sandwich culturing and TUDCA treatment resulted in substantial elevation in the expression levels of ABC transporters playing pivotal role in hepatocytes, such as ABCB11, ABCC3, and ABCC6, as well as the hepatic drug-metabolizing enzymes, CYP1A1 and CYP3A7.

**Fig 5:**
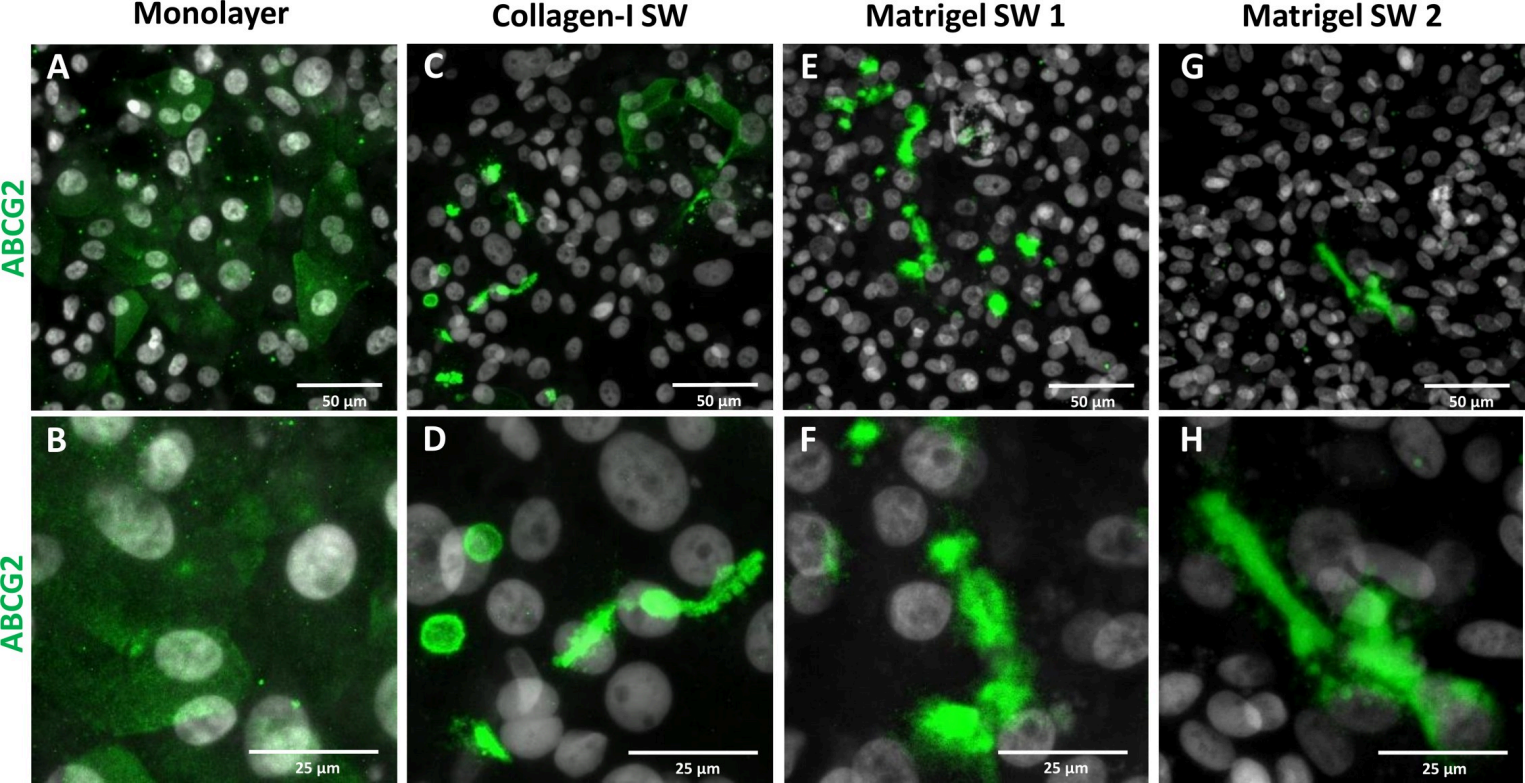
Subcellular localization of ABCG2 transporter in stem cell-derived mature hepatic cells grown under various sandwich culturing conditions. ABCG2 was immunostained (green) and the nuclei were counterstained with DAPI (grey) in the differentiated cells on day 22. The cells were grown on (A) a single Matrigel layer (Monolayer) or (B) overlaid on day 13 with type I Collagen (Collagen-I SW), (C) lower (0.05 mg/mL) or (D) higher (0.25 mg/mL) concentration of Matrigel (Matrigel SW 1 and Matrigel SW 2, respectively). The lower panels (E-H) show the localization at higher magnification. The maximum projections of z-stack of representative confocal images are shown.

Previous studies suggested that sandwich culturing arrangement also can modify the protein subcellular localization in polarized cells [43,44]. Even HepG2 and HepaRG cells could form canaliculus-like structures (semi-canaliculi) under polarizing culturing conditions as demonstrated in S4 Fig. To explore the effect of sandwich culturing on stem cell-derived HLCs, we employed various overlay methods, such as coating the cultures with collagen type I or Matrigel in two different concentrations. In these experiments ABCG2 was used as cell polarity marker, since i) its expression levels in HLCs and adult liver samples were comparable; ii) this membrane transporter in primary hepatocytes is localized to the canalicular membrane under polarizing conditions. In monolayer configuration, the immunofluorescence staining showed that ABCG2 protein was evenly distributed in the HLCs (Fig 5A and 5B). However, under sandwich culturing conditions, the protein was rather accumulated at the cell boundaries in canaliculus-like structures, regardless the applied matrix and its concentration (Fig 5C–5H). Similar results were obtained when another canalicular ABCC2 (MRP2) and the basolateral SLC10A1 (NTCP) were investigated (S5 Fig). These observations suggest that polarizing culturing conditions promote canalicular structure formation and proper localization of transporter proteins in stem cell-derived HLCs.

**Fig 6:**
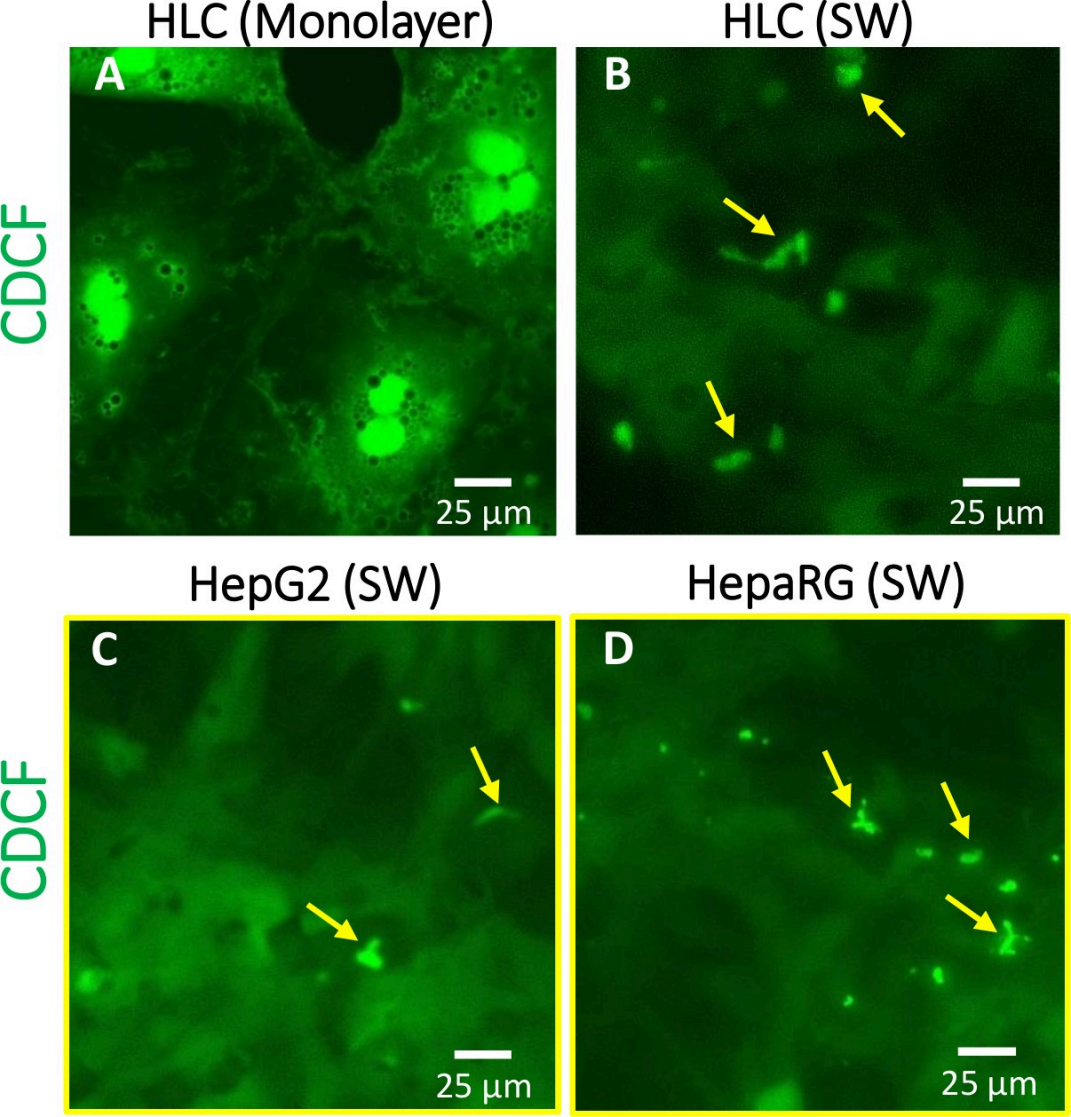
Effect of sandwich culturing on CDCF extrusion in stem cell-derived mature hepatic cells. HLCs grown in monolayer (A) or Matrigel sandwich (B) configuration were subjected to 2μM CDCFDA, and the compartmentalization of the dye was assessed. As controls, sandwich-cultured HepG2 (C) and HepaRG (D) cells were used. The yellow arrows indicate canalicular-like structures.

To explore the functionality of the transporters in polarized or non-polarized cultures, CDCFDA efflux assay was performed with mature HLCs grown in monolayer or sandwich culturing configuration. In monolayer culture of HLCs, the CDCF, the metabolite of CDCFDA, accumulated intracellularly (Fig 6A). However, when the HLCs were grown under polarizing conditions, the dye accumulated in canaliculus-like structures (Fig 6B). As demonstrated in S6 Fig., the tight junction protein, claudin-5 in HLCs grown in monolayer appears at the cell peripheries, whereas in sandwich-cultured HLCs, it is localized in tubular structures, supporting that the formations seen in Fig 6B are likely underdeveloped bile canaliculi. Similar structures were observed, when ABCG2 or MRP2 were stained in HLCs grown in sandwich culturing configuration (Figs 5C–5H and S5B). In the CDCFDA efflux experiments, HepG2 and HepaRG cells grown in sandwich culture configuration were used as controls. In these cell lines, the CDCF appeared in semi-canaliculi, but some intracellular dye accumulation can also be observed (Fig 6C and 6D). The localization of the tight junctions in sandwich-cultured HepG2 and HepaRG cells are shown in S4 Fig. Taken together, the dye efflux assay clearly demonstrates that functional transporters require proper localization, which can be achieved by polarizing culturing conditions.

### Cluster and principal component analysis of expression profiles

**Fig 7:**
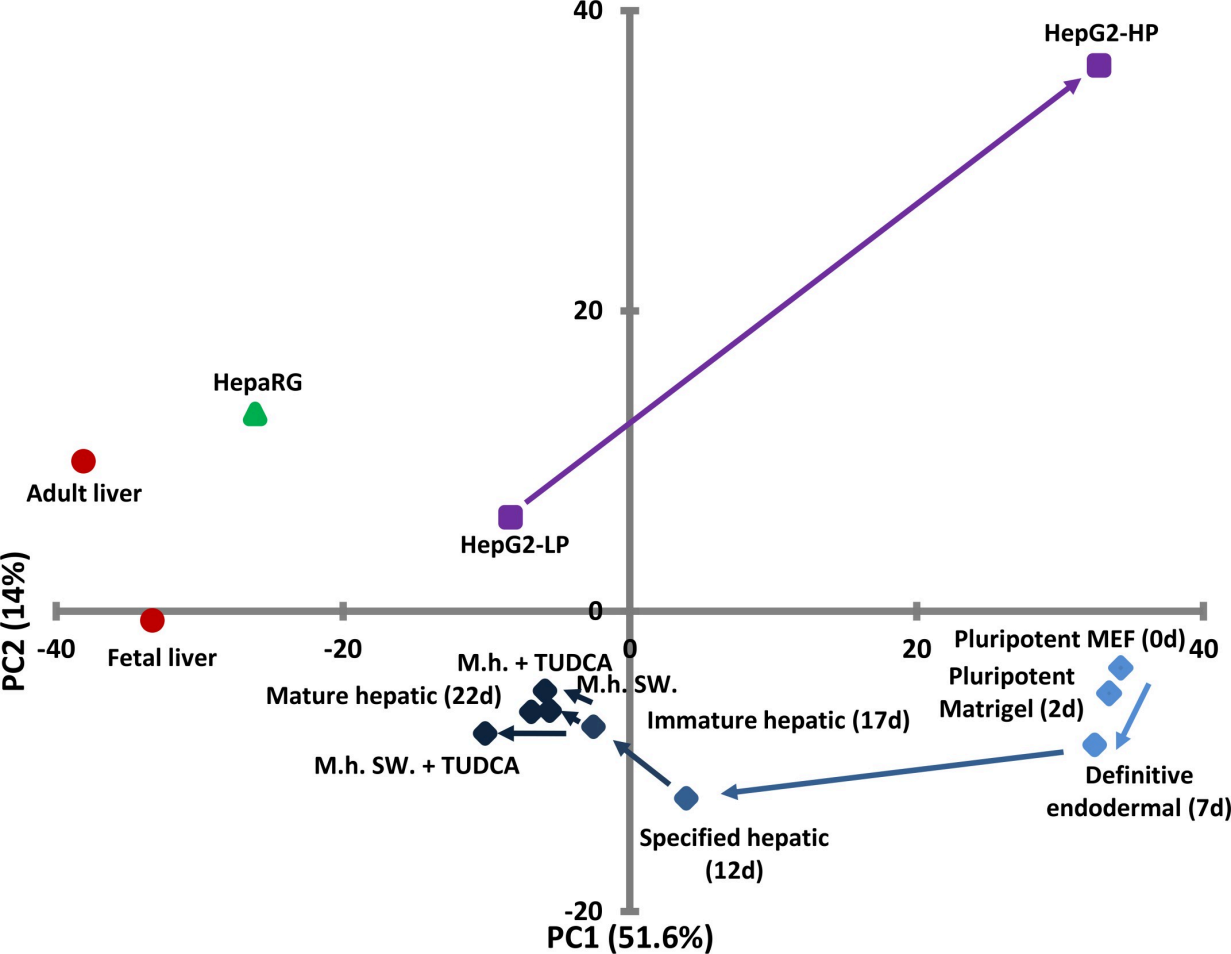
Principal component (PC) analysis of expression profiles of the samples shown in Fig 4. The projection plane was defined by the first two principal components. Related samples are labelled with the same markers and colors. For more details see the legends for Fig 4. The arrows indicate the consecution of samples when applicable.

Based on the mRNA expression profiles assessed by the TLDA, cluster and principal component analyses were performed. One of two major clusters included the pluripotent stem cells either on MEF or Matrigel, and the definitive endoderm stage (Fig 7). Interestingly, HepG2 with high passage (HepG2-HP) was clustered with this group. The other major cluster included the HUES-derived hepatic cells from the specified hepatic to the mature hepatic stages, as well as all the reference samples with the exception of HepG2-HP. HepaRG cells appeared the closest to the fetal and adult liver samples.

Principal component analysis is a powerful tool to reduce the dimensions of complex datasets, including mRNA expression arrays, by finding the underlying structure of the data. The first principal component is a dimension with the highest variance; the second is orthogonal to the first one and has the largest variance possible, etc. In our case the first principal component (PC-1) explains 51.6%, whereas the PC-2 accounts for 14% of the total observed variance. PC-1 is mainly determined by pluripotency and early hepatic marker genes, such as POU5F1, SOX2, and CXCR4, at one end; whereas hepatocyte marker genes, like ALB, SERPINA1 (AAT), and CYP3A7, dominate the other end of PC-1 (S2 Table). PC-2 is mainly determined by transporter genes, such as SLCO1B3, ABCB1, ABCC2, ABCC3, as well as by early hepatic and stem cell marker genes, like AFP, and GATA4 genes. Plotting of each sample among the first two principal components, the progress of differentiation is apparent. Starting from the pluripotent stages appearing on the right side of the PC plot, the differentiated samples gradually move toward the liver tissue samples showing up at the far left. From the reference cell lines, HepaRG cells are localized close to the tissue samples. HepG2 cells with low passage number appear in the vicinity of stem cell-derived hepatic cell; however, the HepG2 with high passage number is positioned far from any other sample, demonstrating the genetic instability of this cell line.

The samples are well-separated among the two principal components because of the distinct expression profiles of the tissue samples and the pluripotent cells. This high variance between these groups determines that the lineage-specific genes have higher loading in the principal components. A few transporter genes appeared to be determinants in PC-2, but several others exhibited relatively low loadings. Although these proteins are essential for numerous hepatic functions, many of them do not contribute significantly to the principal components as a result of being expressed in a number of the samples. This is well exemplified by ABCG2, which is expressed at high level not only in hepatocytes and HepG2 cells, but also in pluripotent stem cells, and after a transient decline also in the differentiated HLCs from the specified hepatic stage throughout the differentiation. Despite its significance in hepatic detoxification, ABCG2 has a low loading in the principal components. These results suggest that mRNA profiling by itself is not satisfactory to judge the goodness of the stem cell derived HLCs.

## Discussion

High quality hepatocyte-like cells are critical for pharmacological and toxicological studies [45,46]. *In vitro* generation of these cells from pluripotent stem cells is a great promise, because pluripotent cells have unlimited proliferative capacity and can be differentiated into any cell type, including hepatic cells. The hepatic differentiation protocols greatly improved in the last decade. In addition to the early spontaneous differentiation methods, multi-step, directed differentiation procedures mimicking the *in vivo* stages of liver development have been established. These protocols were successfully applied with various stem cell types, e.g., embryonic, induced pluripotent, tissue specific stem cells. There is a wide variety of solutions to enhance the liver-specific character of the stem cell-derived HLCs. These include chemically defined xeno-free media [47,48], different coating substances and 3D culturing compositions [35–39], as well as co-culturing with supporting cells [26], bioprinting of HLCs onto structures mimicking *in vivo* situation [49]. It is important to note that the criteria for defining stem cell-derived HLCs were restricted in most cases to the expression of AFP and albumin, secretion of urea and albumin, as well as glycogen storage. Only few studies investigated the metabolizing enzymes, such as cytochromes P450 (CYPs). Despite the high number of studies and the pharmacological relevance of transporter proteins, only negligible attention was paid to the expression and function of membrane transporters in these hepatic model systems.

To fill this gap, in the present study we focused not only on the generally used differentiation criteria listed above, but also on a variety of transporter proteins essential for hepatic functions. Using an efficient differentiation protocol [30], we successfully differentiated HUES9 embryonic stem cells into HLCs. The generated stem cell-derived hepatic cells were functionally active regarding the production of hepatocyte-specific secreted substances, such as albumin and urea. Using a TLDA array specifically designed for combined assessment of gene expressions of various stem cell and hepatic differentiation markers, as well as essential hepatic transporters, comprehensive gene expression profiling were performed throughout the differentiation progress from pluripotent stem cells to differentiated hepatic cells. This analysis demonstrated gradual loss of pluripotency, transient increase of endodermal and early hepatic genes, and progressive gain of hepatic character of the differentiated cells. The typical liver marker genes, such as AFP, albumin, SERPINA1, ASGR1, CYP1A1, were greatly induced, whereas other hepatic marker genes were induced to only a smaller extent. Interestingly, a residual AFP expression, which is rather phenotypic for the fetal liver, was observed in the HLCs even at the mature hepatic stage. Also, some cholangiotic markers, such as KRT7, KRT17, and CFTR, were expressed in these cells. These observations can be explained by the substantial presence of cholangiocytes in the differentiated culture, or by dual character of the cells obtained by the end of differentiation.

It is noteworthy that the hepatic differentiation markers in the stem cell-derived HLCs attained higher levels than found in the widely used hepatic model cell line, HepG2, verifying their profound hepatic character. More importantly, expression of several hepatic genes as well as albumin secretion exhibited substantial decline in HepG2 cells after several passages, reflecting the genetic instability of a cell line of cancerous origin. This implies that their application in pharmacological studies is fairly limited and requires great precaution. As regards the expression profile, HepaRG cells exhibited a better hepatic character, which fell closer to that of the adult liver sample; however, their pharmacological applicability is limited by their restricted growth capacity. Although the stem cell-derived HLCs at the mature hepatic stage practically do not proliferate, as demonstrated by the cell proliferation assay, their cell source, the pluripotent stem cell line possesses unlimited growth capacity. By all means the ceased proliferation is typical for hepatocytes in the healthy liver. Also, the profound presence of binucleated cells in the HLC cultures, which is also a hallmark of hepatocytes, further supports the hepatic character of the differentiated cells.

The transporter composition of the differentiated cells partly reflected that found in the adult liver, although the levels of transporter-coding genes were typically lower than that seen in liver tissue samples. It should be noted that RNA preparations originated from cell cultures are not directly comparable with tissue mRNA samples, especially considering the potential differences in the expression of reference genes. Nevertheless, levels of transporter genes in the stem cell-derived hepatic cells were similar or even higher to that found in the widely-used liver model cells, HepG2 and HepaRG.

To obtain additional information on our HLC cultures, immunofluorescence staining for crucial hepatic proteins was also performed. The presence of the HNF4α and albumin is essential for hepatocytes [50,51]. Our results revealed that these important markers were present in the stem cell-derived hepatic cells. In concert with the results of the mRNA expression study, AFP showed elevated protein expression even at the final stage of the differentiation. It should, however, be noted that the distributions of albumin and AFP in the HLC cultures were not homogenous, which cannot be detected by mRNA expression profiling. This inhomogeneity is typical for the pluripotent stem cell-derived cell cultures, since these cells tend to differentiate into various directions, and directed differentiation only encourages the cells to differentiate toward one particular lineage. This is a recent limitation of the utilization of pluripotent stem cell, and also a challenge for the development of new protocols. Interestingly, ABCG2 is not expressed homogeneously in even stem cells [52], as we observed uneven distribution of ABCG2 in their hepatic progenies (Fig 5). By all means mRNA-based methods are not suitable for unraveling this problem, only immunofluorescence staining can demonstrate the inhomogeneity in the differentiated cultures.

The assessment of keratin 18 and 19, which are hepatocyte and cholangiocyte markers, respectively, demonstrated that some HLCs predominantly express KRT18, some others rather contain KRT19. Also, simultaneous expression of KRT18 and KRT19 can be observed in numerous cells. This bipotential character along with the residual AFP expression is typical for hepatic progenitor cells. Again, this demonstrates that differentiation of pluripotent stem cells results in inhomogeneous cultures, which may contain several cell types. In our case, HLC cultures encompass cells with hepatocyte, cholangiocyte and also dual (potentially hepatic progenitor) character.

Conditioning the HLC cultures with bile acid, namely TUDCA, had only a minor effect on mRNA expression profile. Similarly, sandwich culturing itself did not alter the mRNA expressions of hepatic markers and transporters substantially. However, the combination of sandwich culturing and TUDCA treatment resulted in considerable elevation in the expression of relevant hepatocyte transporters and metabolizing enzymes. More importantly, our experiments on the subcellular localization of transporter proteins revealed the importance of proper cell polarization. ABCG2 was predominantly found intracellularly, when the cells were cultured in regular configuration; however, under polarizing conditions the transporter was correctly localized to the canalicular plasma membrane. Similarly, sandwich culturing promoted proper localization of MRP2 and NTCP transporters to the canalicular and basolateral membranes, respectively. These observations demonstrate that the stem cell-derived HLCs undergo at least partial polarization under sandwich culturing conditions. For pharmaceutically relevant hepatic model systems, the ample expression of the liver transporters is crucial despite the fact that their importance is regularly overlooked [42–44]. It is important to note that the decent expression level of the transporters represents only a necessary condition. Since localization of the transporter proteins in the proper membrane compartment is essential for their normal function, cell polarization is also a key factor for applicable stem cell-derived hepatic models.

Collectively, based on the data presented here, criteria for evaluating the applicability of stem cell-derived HLCs ought to be amended. For comprehensive screenings, mRNA-based assays, such as the TLDA array or RNAseq data, are very useful and can provide wide-ranging, but bulk information on the quality of the cultures. Assessment of expressions at the protein level is more essential, as numerous factors, such as mRNA stability, translation, post-translational modifications, protein stability, etc., all influence the fate of the proteins, thus their steady state levels. Decoupling of mRNA and protein levels especially applies to the membrane proteins [53], which are the key players for hepatic functions determining drug distribution and toxicity. Moreover, our data on subcellular localization of membrane transporters, such as ABCG2, MRP2, and NTCP, demonstrated that normal protein expression level is necessary but not a sufficient criterion; only ample expression coupled with proper cellular localization can confer normal functionality. Correct localization of membrane transporters in hepatic cells, however, requires proper cell polarization, which can be achieved by efficient polarizing culturing conditions, such as sandwich culturing configuration.

The most commonly used hepatocyte markers include AFP, ALB and KRT18; however, assessment of HNF4α and CYP enzymes can also be very informative. Moreover, hepatic transporter proteins, such as ABCC2, ABCC4, ABCC6, ABCB11, and ABCG2, as well as SLC10A1 and SLC47A1 can convincingly confirm the hepatocyte character of the stem cell-derived HLCs. In addition to the above listed hepatocyte markers, the differentiated cultures should also be checked for KRT7, KRT19, and CFTR expression, which indicate the presence of cholangiocytes in the cultures or alternatively dual, hepatocyte/cholangiocyte character of the end-stage cells as demonstrated in our experiments. For generation of well-functioning HLC cultures for pharmaceutical screens, these cholangiocyte markers should be diminished as much as possible by further improving of the differential protocols. A recent attempt include longer maturation periods in the protocol [54], which can unambiguously improve the quality the differentiated cell, however, also increase the costs of the generation of these cultures. It is noteworthy that there is further potential primarily in the maturation phase of the employed differentiation protocols. Resembling the *in vivo* environment even in the maturation phase could further enhance the quality of the HLCs.

As regards the functional assays, assessment of albumin and urea secretion is essential. Periodic acid-Schiff (PAS) staining confirming glycogen storage is also very informative [28,39]. However, ICG uptake and release [28,39,51], or alternatively CDCFDA extrusion assay [51,55] can unambiguously confirm the functional presence of hepatic transporters. Despite the fact the expression levels of canalicular efflux transporters were comparable in monolayer- and sandwich-cultured HLCs, our CDCFDA experiment clearly demonstrated that the dye was extruded to the canalicular structures only when the cells were grown under polarizing condition (Fig 6).

In addition to the essential hepatic transport functions, drug metabolizing capacity of the HLCs is also a key factor, when the cultures are used for pharmaceutical applications. In our study, we assessed the mRNA levels of several hepatic CYPs, and found that CYP1A1, CYP3A7, and CYP3A4 were induced at the final phase of hepatic differentiation of stem cells. The expression level of CYP1A1 in the HLCs was comparable with that of the adult liver sample, whereas the expression of the fetal CYP3A7 and the adult CYP3A4 was about 2 orders of magnitude smaller than in fetal and adult liver samples, respectively. However, CYP1A1, CYP3A7 expressions were substantially increased, when the HLCs were cultured in sandwich culturing configuration and treated with TUDCA. Drug inducibility of CYP enzymes is also an important indicator of drug metabolizing capacity of the hepatic cells; however, this feature is beyond a scope of our recent study. Previously, a few studies investigated the basal and drug-induced activities of CYPs in stem cell-derived HLCs, and demonstrated noticeable CYP activities that were further stimulated by drugs [38,56]. It should, however, be noted that these activities were 1000-fold smaller than found in primary hepatocytes.

In summary, our study provided a comprehensive analysis of human ES cell-derived HLC cultures as regards mRNA expression profiling, expression of key hepatic proteins, their subcellular localization, and critical hepatic functions. Our results demonstrated the significance of membrane transporter proteins in these cultures, and also revealed the importance of cell polarization for generation of well-functioning HLC cultures. In addition, our experiments also call attention to the instability and as a consequence to the limited applicability of the commonly used liver model cell line, HepG2. Previous observations [38,56] and the data presented here suggest that the generally applied criteria for the differentiated HLCs are insufficient. Considering that polarized and functional expression of hepatic transporters is essential for liver functions [2,3,13,15,17,18], we recommend an augmentation of the usual tests with the assessment of the functional presence and proper localization of these transporter proteins when the stem cell-derived hepatic model systems are evaluated.

## Supporting information

S1 Table Relative gene expressions of cells differentiated from HUES9 cell toward the hepatic lineage. In Fig 4, the mRNA expression levels relative to the housekeeping genes were depicted, whereas in this table the fold changes are indicated. In most cases, the end point of the differentiation (mature hepatic stage) was used as a reference point as indicated by yellow highlighting. When a particular gene was not detected in the mature hepatic sample, the expression levels were normalized to that of the fetal liver RNA sample. In three cases HUES9 cells grown on feeder cells, whereas in two other cases, the adult liver RNA sample were used as a reference point as indicated. Red highlights indicate wherever substantial induction were obtained in response to sandwich culturing and/or TUDCA treatment. N.D.–not detected. Detailed descriptions of the studied genes are also listed.
(XLSX)

S2 Table The loadings of each gene expression in the first two principal components. A detailed description of each studied gene can be found in S1 Table.
(XLSX)

S1 Fig Variation in albumin and urea secretion in hepatic model cells. The supernatants from HepaRG cells as well as from HepG2 cultures with different passage numbers (HP–high passage, p > 25; LP–low passage, p < 5) were collected 24 hours after medium change. The concentrations were measured and normalized to the total protein of each sample. The error bars represent S.E.M. (n = 3).
(TIF)

S2 Fig Changes in CYP3A4 expression during hepatic differentiation of HUES9 cells. mRNA expression levels of CYP3A4 were determined at various stages of differentiation. For comparison, the expressions were also assessed in HepaRG, HepG2-HP, HepG2-LP cells, as well as in fetal and adult liver RNA samples. As in S1 Table, the mature hepatic stage was used as a reference point.
(TIF)

S3 Fig Comparison of expression levels of selected genes in HLCs derived from three independent differentiations. The mRNA expression levels of three hepatic markers (A-C) and two ABC transporters (D-E) were assessed by qPCR. HUES-9 cells grown on Matrigel for 2 days (pluripotent), mature (22d) HLCs of three independent differentiation (mature hepatic), HepG2 cells, and adult liver sample were compared. The error bars represent S.D. of the technical replicates (n = 3).
(TIF)

S4 Fig Formation of canaliculus-like structures in sandwich-cultured HepG2 and HepaRG cells. The tight junction protein, occludin was immunostained (green) and the nuclei were counterstained with DAPI (grey) in HepG2-LP and HepaRG cell cultures grown in Matrigel sandwich configuration (0.25 mg/mL). The maximum projections of z-stack of representative confocal images are shown. Yellow arrows indicate semi-canaliculi in partially polarized cultures.
(TIF)

S5 Fig Subcellular localization of NTCP and MRP2 transporter proteins in HLCs. NTCP (green) and MRP2 (red) were immunostained, as well as the nuclei were counterstained with DAPI (grey) in stem cell-derived mature hepatic cells grown under monolayer (A) and Matrigel sandwich (0.25 mg/mL) (B) culturing conditions. The maximum projections of z-stack of representative confocal images are shown. The green and red arrows indicate basolateral and canalicular-like localizations, respectively.
(TIF)

S6 Fig Formation of canaliculus-like structures in differentiated HLCs cultured under polarizing conditions. The tight junction protein, claudin-5 was immunostained in stem cell-derived HLCs grown in monolayer (A) or Matrigel sandwich configuration (B). The nuclei were counterstained with DAPI (grey). The maximum projections of z-stack of representative confocal images are shown. Yellow arrows indicate canalicular-like structures in the polarized cell culture.
(TIF)
