# Supplementary figures and images for: The importance of transporters and cell polarization for the evaluation of human stem cell-derived hepatic cells
Source: PLoS One. 2020 Jan 23;15(1):e0227751. doi: 10.1371/journal.pone.0227751 (PMC6977753; doi:10.1371/journal.pone.0227751)

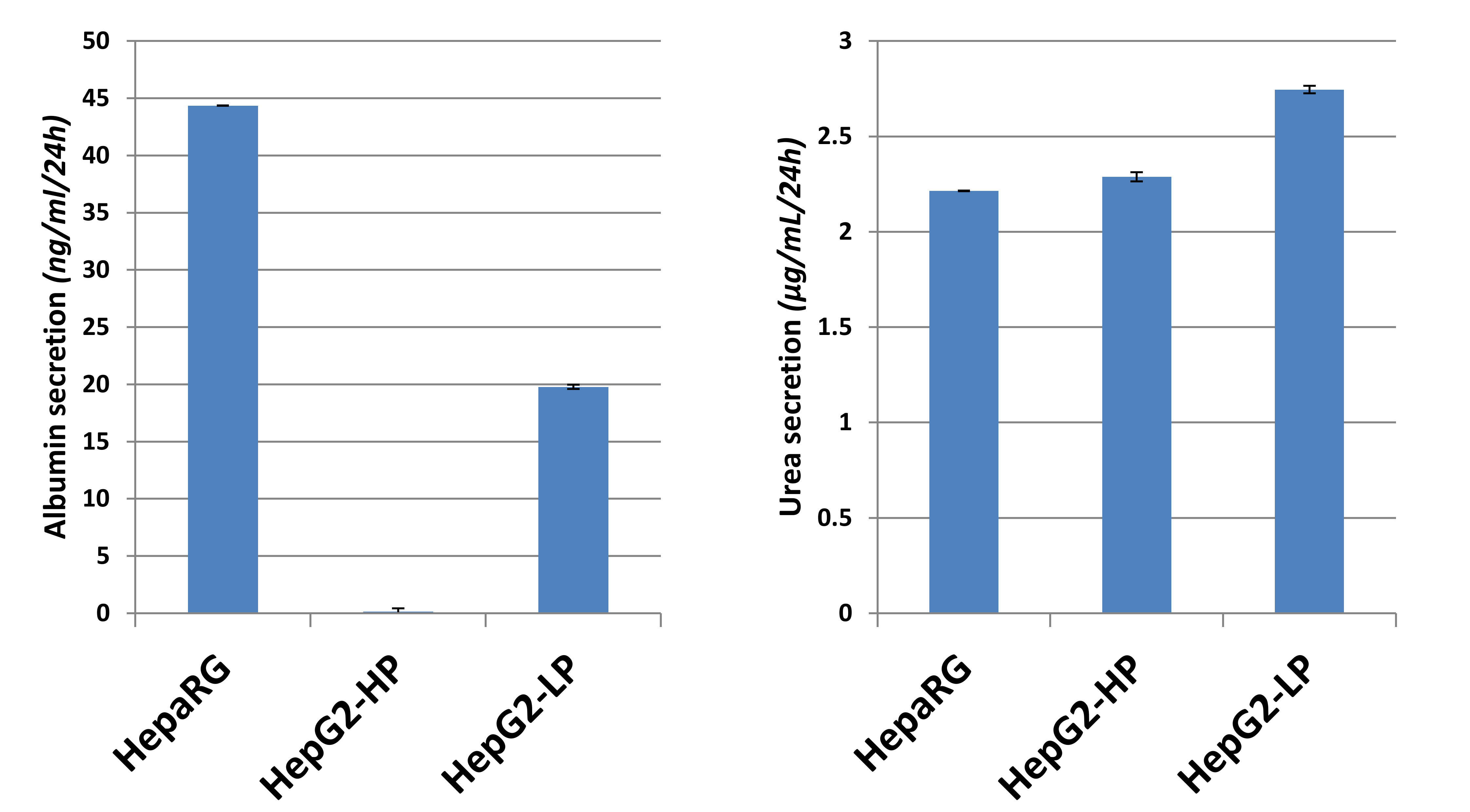

Supplement: S1 Fig — The supernatants from HepaRG cells as well as from HepG2 cultures with different passage numbers (HP–high passage, p > 25; LP–low passage, p < 5) were collected 24 hours after medium change. The concentrations were measured and normalized to the total protein of each sample. The error bars represent S.E.M. (n = 3). (TIF) [file pone.0227751.s003.tif]

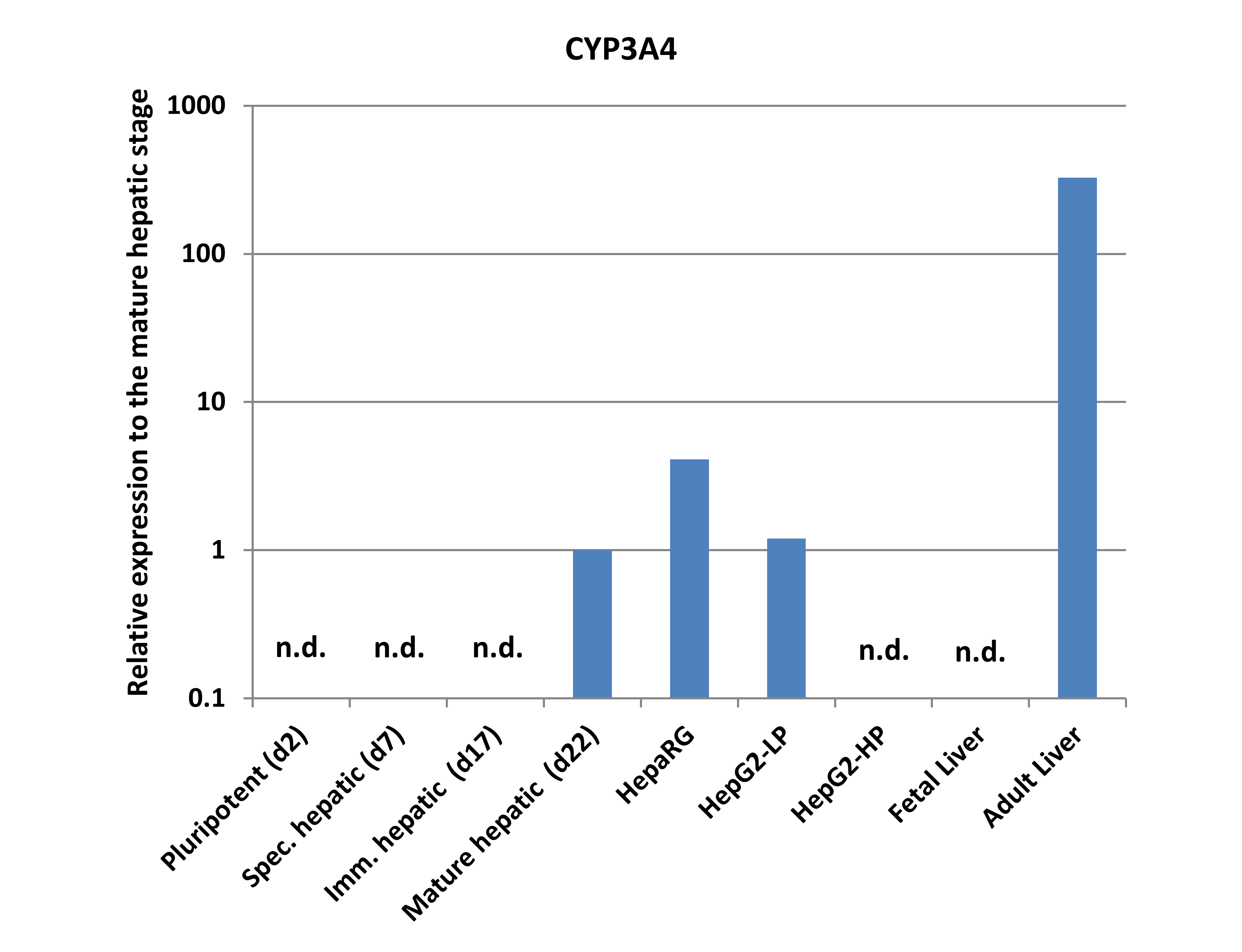

Supplement: S2 Fig — mRNA expression levels of CYP3A4 were determined at various stages of differentiation. For comparison, the expressions were also assessed in HepaRG, HepG2-HP, HepG2-LP cells, as well as in fetal and adult liver RNA samples. As in S1 Table, the mature hepatic stage was used as a reference point. (TIF) [file pone.0227751.s004.tif]

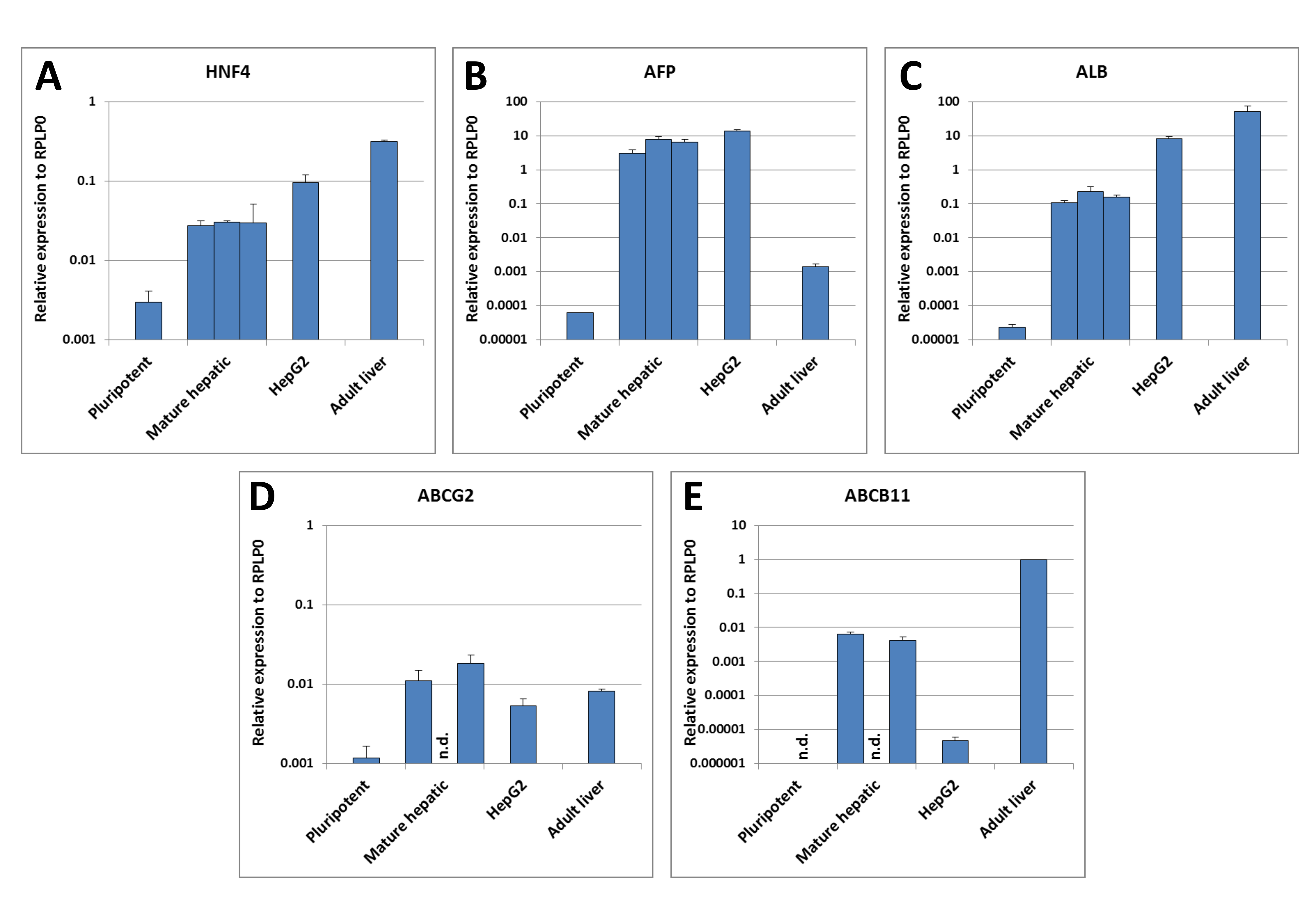

Supplement: S3 Fig — The mRNA expression levels of three hepatic markers (A-C) and two ABC transporters (D-E) were assessed by qPCR. HUES-9 cells grown on Matrigel for 2 days (pluripotent), mature (22d) HLCs of three independent differentiation (mature hepatic), HepG2 cells, and adult liver sample were compared. The error bars represent S.D. of the technical replicates (n = 3). (TIF) [file pone.0227751.s005.tif]

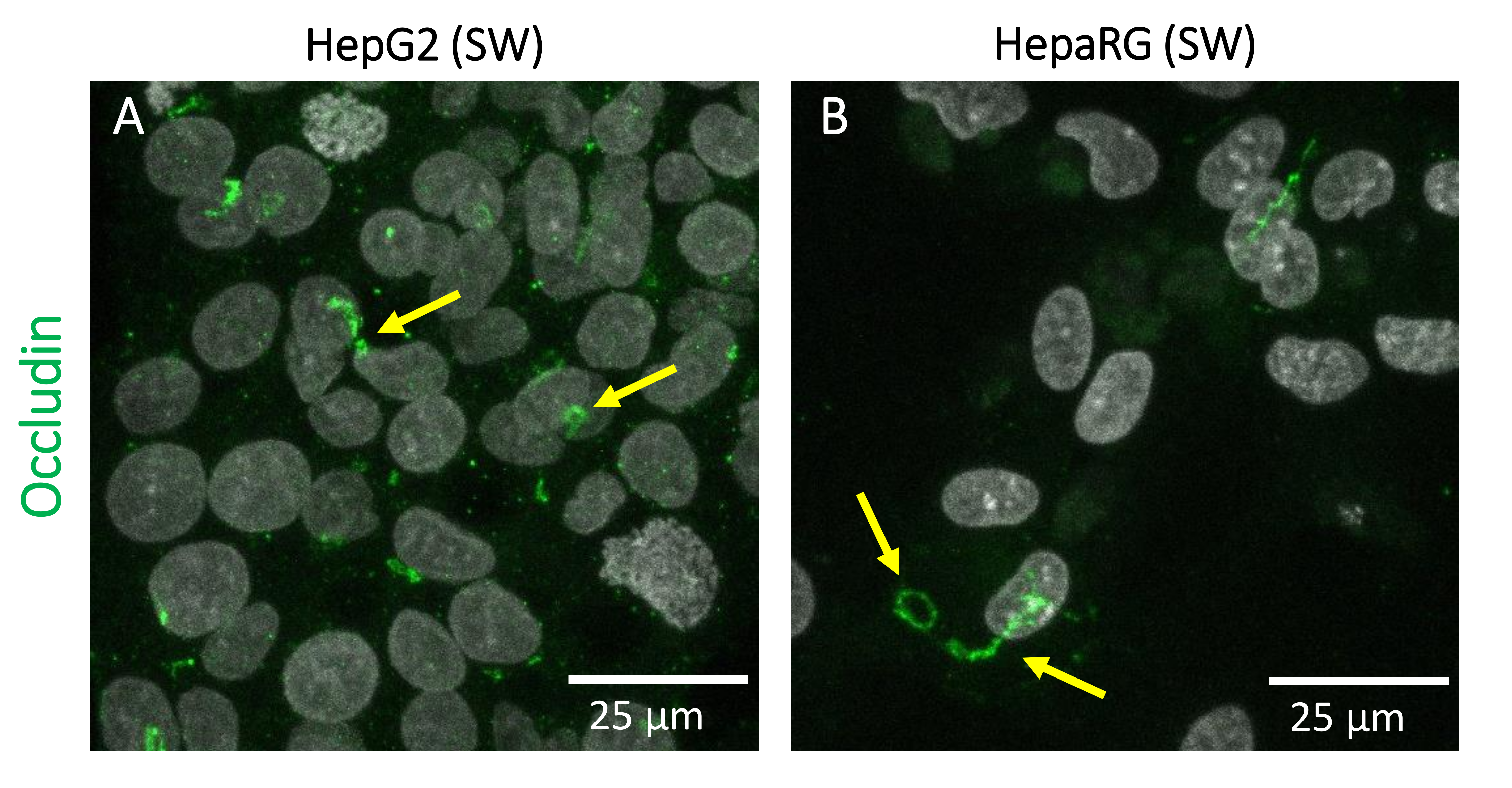

Supplement: S4 Fig — The tight junction protein, occludin was immunostained (green) and the nuclei were counterstained with DAPI (grey) in HepG2-LP and HepaRG cell cultures grown in Matrigel sandwich configuration (0.25 mg/mL). The maximum projections of z-stack of representative confocal images are shown. Yellow arrows indicate semi-canaliculi in partially polarized cultures. (TIF) [file pone.0227751.s006.tif]

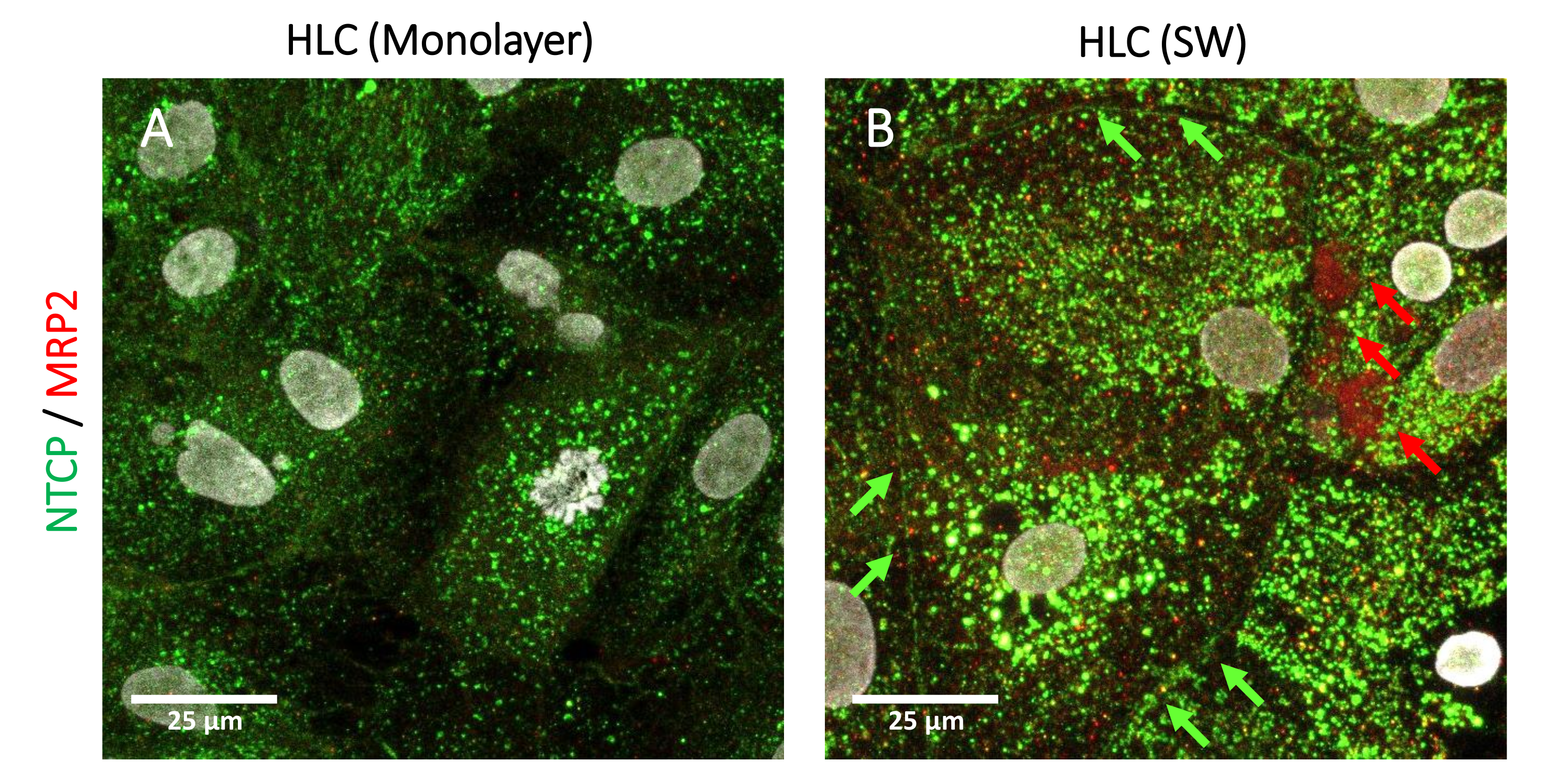

Supplement: S5 Fig — NTCP (green) and MRP2 (red) were immunostained, as well as the nuclei were counterstained with DAPI (grey) in stem cell-derived mature hepatic cells grown under monolayer (A) and Matrigel sandwich (0.25 mg/mL) (B) culturing conditions. The maximum projections of z-stack of representative confocal images are shown. The green and red arrows indicate basolateral and canalicular-like localizations, respectively. (TIF) [file pone.0227751.s007.tif]

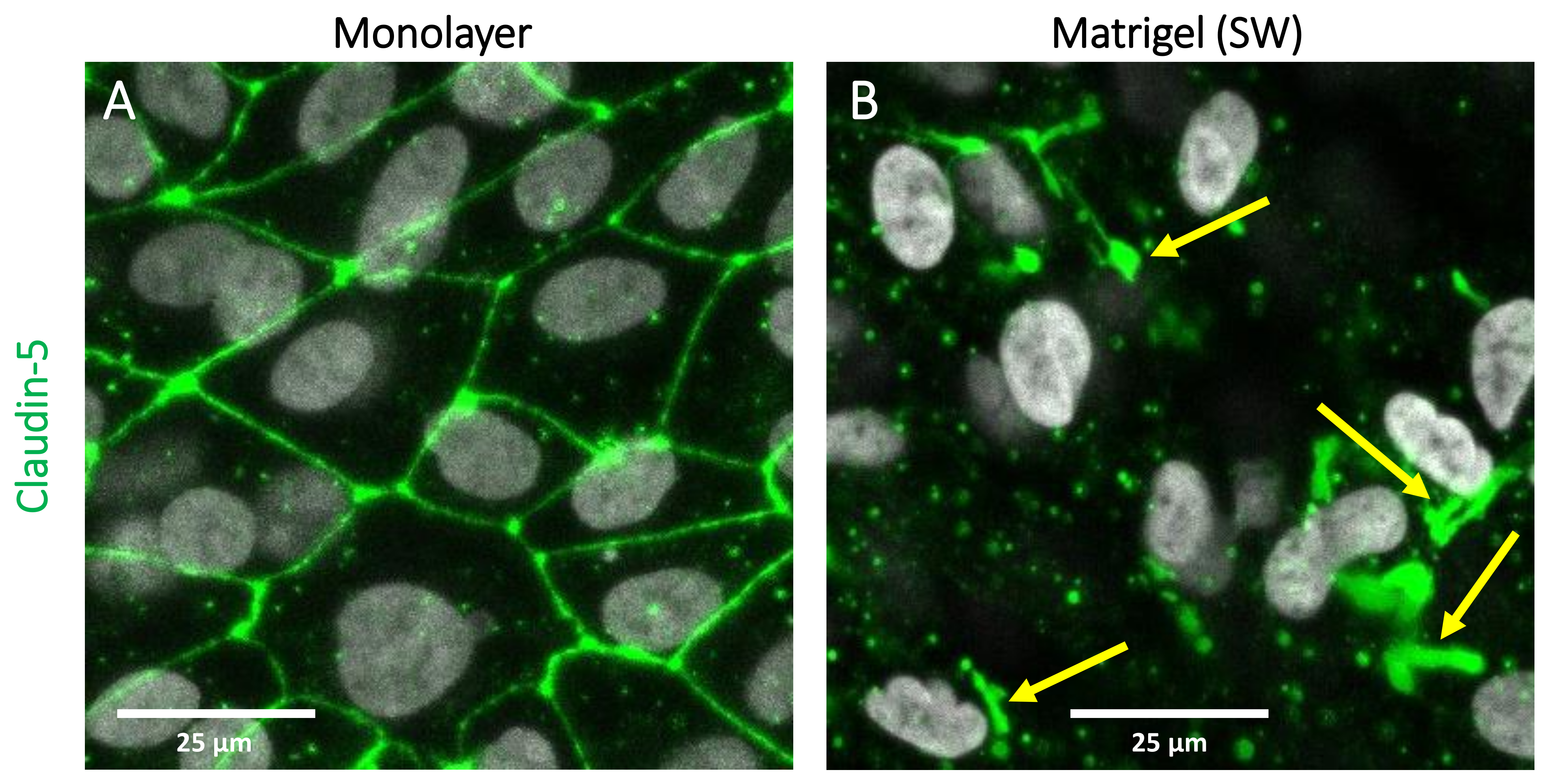

Supplement: S6 Fig — The tight junction protein, claudin-5 was immunostained in stem cell-derived HLCs grown in monolayer (A) or Matrigel sandwich configuration (B). The nuclei were counterstained with DAPI (grey). The maximum projections of z-stack of representative confocal images are shown. Yellow arrows indicate canalicular-like structures in the polarized cell culture. (TIF) [file pone.0227751.s008.tif]
